# Negotiating research in the Emergency Department: a qualitative study of staff experience of the Distal Radius Acute Fracture Trial CAst versus SPlint (DRAFT3-CASP) RCT for distal radius fractures

**DOI:** 10.1302/2633-1462.75.BJO-2025-0374.R1

**Published:** 2026-05-19

**Authors:** Emma E. Phelps, Elizabeth Tutton, Jenny Gould, Liz Baird, Juul Achten, Matthew L. Costa

**Affiliations:** 1 Kadoorie Institute for Trauma, Emergency and Critical Care (NDORMS), University of Oxford, Oxford, UK; 2 Major Trauma Centre, Oxford University Hospital NHS Foundation Trust, John Radcliffe Hospital, Oxford, UK; 3 Patient and Public Involvement and Engagement (PPIE) partner, Oxford, UK

**Keywords:** Qualitative, Trauma, Trial, Distal radius fracture, Randomized controlled trial, Emergency care research, Orthopaedic injury, Interview, fracture of the distal radius, Acute Fracture, Distal Radius, Randomized Controlled Trials, orthopaedic trauma, plaster cast, wrist splint, fast, clinicians, splint

## Abstract

**Aims:**

We sought to explore staff experience of conducting an orthopaedic trauma randomized controlled trial (RCT) in the Emergency Department (ED). The Distal Radius Acute Fracture Trial: CAst versus SPlint (DRAFT3-CASP) RCT explores the effectiveness of two treatment pathways for patients with a fracture of the distal radius that does not require manipulation. These are, a plaster cast which is removed four to six weeks later in a fracture clinic, and discharge from emergency care with a wrist splint that patients remove themselves.

**Methods:**

A total of 20 multidisciplinary staff recruiting to the DRAFT3-CASP RCT from 14 NHS trusts across England took part in a telephone/online qualitative interview. Interviews were informed by Heideggerian Phenomenology and data were analyzed using thematic analysis.

**Results:**

The findings highlight the struggle to undertake research in emergency settings through the overarching theme ‘negotiating research’. Staff identified three enablers of research: 1) fitting within clinical practice; 2) finding meaning; and 3) being supported. The fast pace of work and high service demand in emergency care prevented clinical staff from fully engaging with the study. Research delivery staff were vital and supported screening, recruitment, and data collection. Finding meaning in the research question by linking it to patient benefit helped staff to maintain enthusiasm despite the challenges encountered.

**Conclusion:**

Negotiating research in the ED is challenging. Protected time is essential for clinical staff to undertake research training and recruitment. Research delivery teams with research expertise are vital for successful recruitment. Increased investment in research delivery staff can ensure patients are given the opportunity to take part in research studies and can promote enthusiasm for research.

Cite this article: *Bone Jt Open* 2026;7(5):667–673.

## Introduction

Recruiting patients to randomized controlled trials (RCTs) places additional demands on clinical staff. To recruit a patient to a trial, clinicians need to identify potentially eligible patients, communicate the trial to patients, and collect baseline data, while completing their usual role and responsibilities.^1^ In orthopaedic trauma research, challenges include clinician and patient treatment preferences, staff availability, and varying interpretation of the inclusion criteria and interventions.^1-4^ There is little published research about staff experience of recruiting to adult orthopaedic trauma trials in Emergency Departments (EDs). However, paediatric orthopaedic trauma trials highlight the role of clinical and research leadership,^5^ and tensions between emergency care and orthopaedic specialities.^4^ In adults, we need to know more about how to enable recruitment in emergency care, particularly where clinical staff are directly involved in recruiting patients.

The Distal Radius Acute Fracture Trial CAst versus SPlint (DRAFT3-CASP) is a multicentre RCT recruiting across the UK.^6^ DRAFT3-CASP compares the clinical and cost effectiveness of a removable wrist splint compared with a plaster cast for distal radius fractures that do not require a manipulation. Patients are recruited to the trial in the ED or a minor injury unit (MIU) at any time of the day or night. Successful recruitment is therefore reliant on staff screening and recruiting to the study in the ED where there is a continuous high demand for the service. The study aimed to identify staff experience of the enablers and inhibitors to research to aid recruitment to the DRAFT3-CASP study and future ED research studies.

## Methods

### Sample

A purposive sample of 20 staff (eight female, 12 male) from 13 NHS trusts recruiting to the trial took part in a qualitative interview via telephone or digital audio/visual media. Purposive sampling was used to achieve a sample which included a range of staff roles, sites spread geographically across England and Wales, and sites with high, low, and average recruitment. Staff from 16 sites recruiting to DRAFT3-CASP were invited to take part in an interview. For eight sites, one member of staff took part; in three sites, two members of staff took part; in two sites, three members of staff took part, and three sites did not respond to the invitation. Sites varied in their recruitment to the study. Across all study sites, average recruitment is 1.3 patients per month. Of the sites taking part in this interview study, four sites recruited on average less than one patient per month, six sites recruit on average between one and two patients per month, and three sites recruited more than two patients per month.

All staff taking part in an interview were actively involved in the DRAFT3-CASP study (nine research nurses and 11 clinical staff; two physiotherapists, five consultants in emergency medicine, one consultant in Trauma & Orthopaedics (T&O), two advanced clinical practitioners and one emergency nurse practitioner (ENP), a specialist nurse trained to assess, diagnose, and treat minor injuries or illnesses). The term ‘staff’ is used to refer to interview participants throughout the results to ensure anonymity. The term ‘research staff’ refers to those members of the study team who were employed specifically to facilitate research delivery. The term ‘clinical staff’ is used to refer to staff within the department who provided the frontline clinical care for patients. All staff received an information sheet and had at least 24 hours to consider their participation. Verbal informed consent was recorded and witnessed by an administrator trained in good clinical practice.

### Interviews

Drawing upon Heideggerian phenomenology,^7^ this study sought to explore each participant’s experience of the DRAFT3-CASP study in the context of their everyday clinical practice. Interviews were semistructured to include key topics such as study setup, screening and recruitment, equipoise, and communication. Questions included ‘can you tell me about your experience of the DRAFT3-CASP study?’, ‘what do you think about the two different patient pathways?’, and ‘what was it like recruiting patients to the study?’ A female researcher (EEP) with a PhD, experienced in health services research and research with staff involved in orthopaedic trauma trials conducted the interviews between September 2023 and May 2024, and they took up to 35 minutes.

### Analysis

Interviews were audio recorded, transcribed verbatim, and data were managed using NVIVO v. 12 (QRS Warrington, UK). Analysis was inductive and data were coded line-by-line.^8^ Codes were grouped together based upon meaning to develop categories and then themes. For example, the codes ‘providing evidence’ and ‘easy to implement’ were grouped into the category ‘implications for practice’. ‘Implications for practice’ was linked with the category ‘patient perspectives’ to create the theme ‘finding meaning’. Analysis was iterative, with new codes added and existing codes revised as more data were analyzed. Rigour and trustworthiness^9^ were achieved through immersion in the data, regular reflection and discussion of the data and developing themes with ET, the inclusion of illustrative quotations to enable readers to consider the interpretation of the data and descriptions of the participants, methods, and context. Data saturation, when no new categories or themes arise from subsequent data collection, was achieved for each theme. The consolidated criteria for reporting qualitative research (COREQ) guidelines informed this article (Supplementary material).^10^

### Patient and public involvement and engagement

Two patient and public involvement and engagement (PPIE) co-investigators have been involved throughout the DRAFT3-CASP study, contributing to the study conception and design, development of the study materials, interpretation of qualitative findings, and are part of the trial management and steering committees. They have contributed to the development of this article and are co-authors.

The DRAFT3-CASP study is registered with the International Standard Randomized Controlled Trials Number Registry (ISRCTN66692543). The South West Frenchay Research Ethics Committee approved the DRAFT3-CASP study, and this embedded qualitative study (REC reference 22/SW/0177).

## Results

**Fig. 1:**
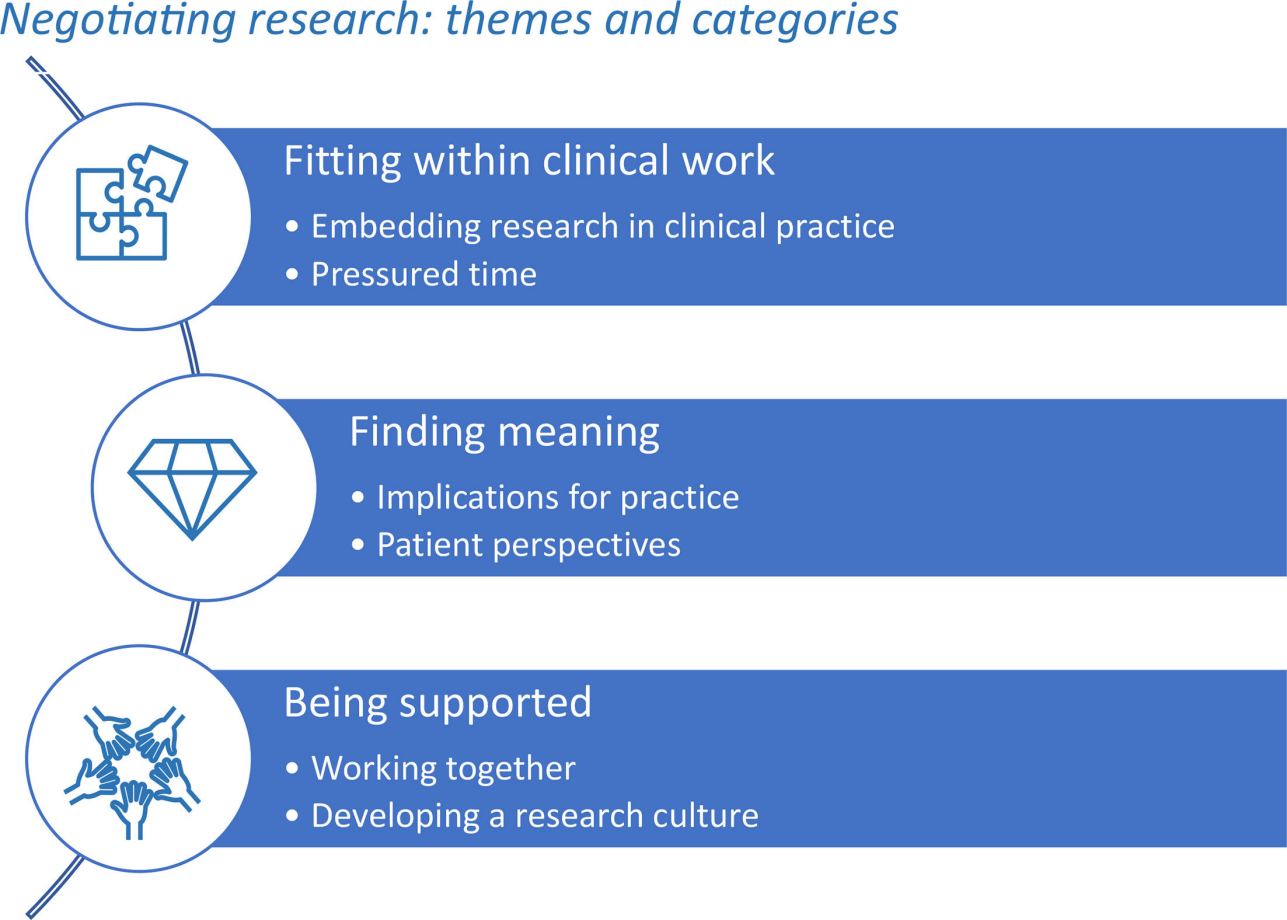
Negotiating research in the Emergency Department: themes and categories.

This study identified the overarching theme ‘negotiating research’ which describes the struggle to engage staff and sustain recruitment in the ED, as shown in Figure 1. Staff identified three enablers of research 1) fitting within clinical practice; 2) finding meaning; and 3) being supported.

### Theme 1: Fitting within clinical work

Fitting within clinical work conveys the importance of ensuring the study can be embedded within clinical practice where identifying patients is challenging and time is pressured. Table I highlights activities to support staff undertaking research in ED from theme 1. Illustrative quotations for theme 1 are provided in the Supplementary Material.

**Table I.** Activities to support staff undertaking research in the Emergency Department (ED) from theme 1.

Be mindful of clinical pressures. Identify a quiet and private place to recruit to the study. Consider different methods for identifying potential. patients (computer systems, different staff involved). Consider patient pathways. Minimize data collection. Streamline trial processes.

ED, Emergency Department.

Category: Embedding research within clinical practice: To engage clinical staff in research in the ED, the study needs to fit with clinical practice. As staff need to include research activities within their existing workload, the ideal would be minimal data collection for clinical staff, and the ability to randomize patients using existing systems without the need to remember additional websites and passwords.

*“To date, my experience from trying to run ED research is that if you want out of hours recruitment, or you want clinician-led and delivered randomizations, it has to be an exceedingly swift, very limited process with predominantly retrospective data collection, and then you’ll get randomization.” (Staff 10)*

The ease with which staff could identify potential patients for this study, varied across sites. Clinical staff endeavoured to identify potential patients, but they struggled with a high patient demand and competing clinical tasks. In some sites, research delivery teams were helped by access to patient lists or computer software that would flag potential patients on admission.

*“I think our challenge is not being able to screen our own patients and so we’re purely relying on our ED and if they’re really, really busy you can understand them forgetting a couple of times.” (Staff 3)*

*“We have what we call EPR (electronic patient records) where we can see all the patients coming into minors (minor injury service) and so we can see them as they come through. A couple of members of the research team will be keeping an eye on that list as it updates, it updates every thirty seconds, and we can see as soon as someone comes in. None of us do nights, but we do weekend shifts and so we might miss people at night but there’s always someone keeping an eye on it.” (Staff 5)*

Changing normal practice, such as not referring patients to fracture clinic for the splint arm of the trial, was challenging, although it became easier with experience. Staff who referred patients to fracture clinic often did so out of habit. But, on some occasions, staff wanted their patients’ radiograph to be reviewed in fracture clinic as a safety net to ensure the fracture was managed correctly.

*“A lot of them (colleagues) felt that with the elderly population it felt hard to send them away without the fracture clinic follow-up. I do think we’ve got wedded to that idea, that they get seen by the specialist, we’re the non-specialists (ED staff), there is that safety net and that comfort blanket of another person seeing them in case we haven’t quite managed it right.” (Staff 13)*

Category: Pressured time and space: A lack of allocated time for research and fast paced, high service demand, hindered clinical staff from engaging with and recruiting to the study. Recruiting a patient to the study took at least 20 minutes and, sometimes up to an hour. This was considered a long time to be away from clinical work, and longer in comparison to other research studies in the ED. Time was often taken completing baseline data collection. This was initially collected prior to randomization but changed to allow patient care to progress in a timely manner. Staff supported this change as it allowed clinical staff to move on to the next patient or enabled the patient’s treatment to be arranged while the baseline data were collected.

*“With DRAFT3-CASP, the downside is the prerandomization questionnaire, no clinician is going to engage with that because you could have moved on to another patient by then, in terms of efficient use of time.” (Staff 10)*

Research teams were aware that clinical staff often struggled with their workload, and it was difficult to ask them to do more. They understood that seeing patients is the priority for clinical staff with research seeming *“*a bit of a luxury*”* (Staff 7). Staff acknowledged the lack of incentive for clinical staff, particularly ENPs, to get involved in research. The responsibility for, and allocation of time for research, was not formalized within their role. Staff appreciated ENPs may be reluctant to recruit patients to research studies when they already felt overworked. Sometimes clinical staff including ENPs were too busy and simply forgot to recruit to the study while at other times they prioritized seeing patients.

Lack of space was also a challenge in some sites. Staff needed somewhere to talk to patients about the study and collect baseline data privately, but this could take space needed for the next patient.

*“There’s not a delegated space that a patient could be taken to privately and so you end up in a cubicle, that probably already needs to be used for something else, while she (research staff) goes through the questions quickly. So that’s the biggest concern from us.” (Staff 10)*

### Theme 2: Finding meaning

Finding meaning conveys the value staff attribute to the study and the implications for their own practice and patient care, with patients’ interest in the study reinforcing the importance of the research question. Table II highlights activities to support staff undertaking research in the ED from theme 2. Illustrative quotations for theme 2 are provided in the Supplementary Material.

**Table II.** Activities to support staff undertaking research in the Emergency Department (ED) from theme 2.

Involve and engage ED clinical staff early in the study development. Enable a sense of ownership - keep staff updated, show relevance and meaning for their practice. Provide research training with the opportunity to reflect upon implications/importance for practice. Involve Patient and Public Involvement and Engagement (PPIE) groups in research awareness to highlight the patient’s perspective. Ensure patient-facing materials are easily available for staff to give to potential participants, i.e posters, leaflets.

Category: Implications for practice: Staff believed the research question was important. They conveyed their enthusiasm and explained their motivation for recruiting to the study to clinical staff. The study was considered valuable, as staff believed it would provide evidence for best practice, and the results would be easy to implement. The study also used patient-reported outcomes allowing practice to be informed by patient outcomes rather than individual clinical judgements. These factors gave their involvement in the study meaning. Staff compared DRAFT3-CASP with other studies where the findings were more likely to benefit another speciality. Staff noted the need to engage clinical staff throughout the study, keeping them up to date, helping them feel a sense ownership, and see the study through to the results.

*“It goes back to that thing ‘meaning’ and I know it sounds really stupid but that’s the crux of getting research into ED. It’s got to mean something just beyond we’re helping another speciality. It’s got to mean something to our patients directly, to our processes directly, to us as carers directly.” (Staff 16)*

Category: Patient perspectives: Patients’ willingness to participate reinforced staffs’ belief in the study. Patients were interested in the study and happy to help, although some had a treatment preference. Differences in patients’ preference emphasized the need for evidence to inform patients of the benefits of both treatments reminding staff not to assume everyone will want a splint.

*“We’ve had both requests from patients, and so some people have opted for a cast, and some people have opted for a splint. Which, I think, has highlighted to the ENP team, that not everybody has the same opinion, not everybody does just want a splint, but yes, having that evidence will help us to be able to communicate to patients in the future. If they are choosing one or the other, we will be able to, certainly with confidence, tell them what will be best.” (Staff 1)*

### Theme 3: Being supported

Being supported conveyed the importance of clinical and research teams working together to successfully recruit to the study and developing a research culture where research was actively supported and promoted. Table III highlights activities to support staff undertaking research in ED from theme 3. Illustrative quotations for theme 3 are provided in the Supplementary Material.

**Table III.** Activities to support staff undertaking research in the Emergency Department (ED) from theme 3.

Find ways to help and lessen the burden on clinical staff. Frequent reminders to keep the study in mind but without pressure. Offer support and training to gain experience and confidence with trial processes and recruitment. Offer opportunities to shadow research staff. Offer peer support or mentorship to clinical staff. Support baseline data collection. Provide protected time and opportunity to complete research training. Engage staff across the department to develop research awareness. Provide staff with evidence of their contribution to use in their professional practice portfolio.

ED, Emergency Department.

Category: Working together: To ensure successful recruitment, staff needed to work together and share research and clinical expertise. Support from research delivery staff was crucial, and many sites struggled to recruit outside the research team’s working hours. Research teams supported screening, informed consent discussions and data collection. They maintained visibility of the study by frequently visiting the ED and reminding staff to screen patients, while being mindful not to pressure staff.

*“So, the research team they’re good actually and they do cover quite a long day, and so, the majority of patients are getting screened by them. They’re normally quite easy to contact, they come down quite willing and are helpful getting them in (patients in the study).” (Staff 14)*

Ensuring all staff within the department were aware of the study enabled early identification of patients. Once patients were seen by clinical staff, treatment and discharge could be swift and patients could be missed.

*“Most distal radial fractures are easy; non-manipulative distal radial fractures are very easy, quick within the department to be seen, sorted, and discharged within minutes. So, as a group of patients trying to capture them (before they are discharged) is the hardest bit.” (Staff 10)*

Without prior research experience and dedicated time for research and training, recruitment was a challenge. Knowledge and expertise in research and research processes such as screening, informed consent, conveying equipoise, and data collection were required. Research delivery staff supported clinical staff to overcome these challenges.

Baseline data collection was time-consuming and difficult for clinical staff. The length of the questionnaire, up to 70 questions, and the repetition for pre and postinjury, could frustrate or confuse staff and patients. Experienced research staff guided patients through questionnaires, found ways to keep patients engaged, and knew which questions were likely to be most difficult.

*“As I said the process is quite long-winded, there is a lot of repetition of questions. Which I think, has infuriated a couple of our patients and staff, and so I would often take over. I would calm down the patient and just explain. Or, for a staff member who’s getting frustrated I’ll tell them to go and see another patient and I’ll finish off.” (Staff 7)*

In some sites research teams provided training for clinical staff, particularly ENPs, to help them recruit to the study and to ensure clinical staff were learning and getting something in return for their effort.

Category: Developing a research culture: Staff described the advantages of working in a department with a strong research culture, where there were ongoing research studies and a variety of staff involved in research.

*“By having a portfolio of different research projects that apply to different patient cohorts, it actually spreads the workload out. It means there’s an equity across different patient groups, to get involved in research, and it doesn’t become such a burden on the department as a whole. Doing an orthopaedic one, like this, is just another string to the bow, I suppose.” (Staff 16)*

In departments where research was supported and embedded, research and clinical teams were integrated, working towards a common goal, shared ownership of the research, and there was support for the time used for recruitment and research training. Without support for research within their department, staff worried there would be consequences for recruiting a patient when they could have seen two patients in that time.

*“I think getting our ENPs on side, getting them involved, getting them engaged. Saying we’re going to target you to be trained up to be the group who are going to lead on this study. I think, that’s really good because they’re the group who are most likely to see the cohort of patients. Also, because they’re a group who haven’t hugely been involved in research projects that we’ve done in the department previously. So, it’s a really positive impact for the integration of a team, by saying we’ve got one for you guys.” (Staff 11)*

## Discussion

The findings highlight the struggle to undertake research in EDs with a high service demand. Despite NHS ideals to embed research into clinical practice,^11^ further organizational support is required.

In this study, staff highlight the negotiation that they undertake to engage clinical staff in research. Enablers were fitting the research within clinical practice, finding meaning in undertaking the research by linking it to patient benefit, and being supported. Successful negotiation has enabled the trial to recruit 1,066 patients to date, from 45 UK EDs and MIUs.

Fitting this study within the existing patient pathway and ED environment was a challenge, as noted in other studies.^1,4,5,12^ While it was anticipated that clinical staff, particularly ENPs, would recruit to the study, most sites relied on research delivery teams. Therefore, recruitment was often unachievable outside of the research teams’ working hours. The findings demonstrate that ENPs need more time and training to recruit to research studies. Recruitment was best in centres where clinical staff felt enabled to lead on recruitment themselves. Furthermore, in this study, changing usual practice by discharging patients directly from the ED without fracture clinic follow-up was challenging. Fracture clinic was perceived as a safety net, ensuring each patient would be reviewed by a specialist.

Increased investment in research delivery teams including teams resourced for out of hours working can support research in EDs, build research cultures within departments, and allow more patients to be offered the opportunity to take part in research. Time and research expertise are needed to ensure patients receive a quality interaction, are given appropriate time to discuss research, understand risks, benefits and what participation involves, ask questions, and give informed consent.^13,14^

Fostering enthusiasm for research through integrated research teams, creative roles that combine clinical work and research, different ways of delivering research training, research questions valued by staff, offering opportunities for research mentorship, and shadowing and developing emergency care research networks could all support clinical staff to become more involved in research. Integrated research teams with expert research delivery staff recruiting to multiple studies, and an awareness of the need for research knowledge and education have contributed to a research culture and an enthusiasm for research in orthopaedic trauma.^1^

The study highlights staff experience of negotiating research in an emergency care context providing direction for future studies in ED. The purposive sample of staff included a range of experience from clinical and research delivery staff in 13 NHS hospitals and one MIU, from across England and included staff from high, average, and low performing sites. PPIE partners and clinical staff express resonance with these findings. Interviews with clinical staff who were not involved in research recruitment, especially ENPs, and sites not recruiting to this study, may identify further barriers and facilitators to research in ED.

In conclusion, negotiating research in an ED is challenging. Protected time is essential for clinical staff to undertake research training and recruitment. Research delivery teams with research expertise are vital for successful recruitment. Increased investment in research delivery staff, and joint research-clinical roles, can ensure patients are offered the opportunity to take part in research studies and can promote enthusiasm for research.

**Take home message**

- Recruiting patients to research studies in Emergency Departments relies on research delivery staff who share expertise, fit research within practice, and convey the value of research.

- To enable clinical staff to fully engage with research, support, research education, and protected time for recruitment are essential.

## Data Availability

The data that support the findings for this study are available to other researchers from the corresponding author upon reasonable request.
